# Prevalence of dementia in mainland China, Hong Kong and Taiwan: an updated systematic review and meta-analysis

**DOI:** 10.1093/ije/dyy007

**Published:** 2018-02-12

**Authors:** Yu-Tzu Wu, Gemma-Claire Ali, Maëlenn Guerchet, A Matthew Prina, Kit Yee Chan, Martin Prince, Carol Brayne

**Affiliations:** 1 Department of Public Health and Primary Care, University of Cambridge, Cambridge, UK; 2 Centre for Global Mental Health, Health Service and Population Research, London, UK; 3 Centre for Global Health Research, University of Edinburgh, Edinburgh, UK; 4 School of Public Health, Peking University Health Sciences Centre, Beijing, China

**Keywords:** Dementia, prevalence, China, Hong Kong, Taiwan, meta-analysis

## Abstract

**Background:**

There are several existing systematic reviews of prevalence of dementia for mainland China, Hong Kong and Taiwan, but several studies have been newly reported. The aim of this study is to update prevalence data in this region and test for variation across geographical areas and time periods using the new dataset.

**Methods:**

Twenty prevalence studies identified from *World Alzheimer Report 2015* (January 2011–March 2015) and an updated search (March 2015–February 2017) were added to the original dataset (*N* = 76). Meta-regression was used to investigate geographical variation and time trends, taking methodological factors and characteristics of study population into account, and to estimate prevalence and number of people with dementia by geographical area.

**Results:**

Compared with northern China, the prevalence of dementia was lower in the central China [-1.0; 95% confidence interval (CI):−2.2, 0.3], south China (−1.7; 95% CI: −3.1, −0.3), Hong Kong and Taiwan (−3.0; 95% CI: −5.0, −1.0) but appeared to be higher in western China (2.8; 95% CI: 0.1, 5.5) after adjusting for methodological variation. The increasing trend from pre-1990 to post-2010 periods was considerably attenuated when taking into account methodological factors and geographical areas. The updated estimated number of people with dementia in all these areas is 9.5 million (5.3%; 95% CI: 4.3, 6.3) in the population aged 60 or above.

**Conclusions:**

Geographical variation in dementia prevalence is confirmed in this update, whereas evidence on increasing trends is still insufficient. Differing societal development across areas provides an opportunity to investigate risk factors at the population level operating across diverse life course experiences. Such research could advance global primary prevention of dementia.

**Key Messages**

- This review incorporates 96 prevalence studies of dementia in mainland China, Hong Kong and Taiwan from previous systematic reviews, and an updated search including both English and Chinese literature published until February 2017.
- The decreasing prevalence of dementia from northern, central and southern China to Hong Kong and Taiwan was confirmed in this update. A high prevalence in western China was identified in this new analysis.
- The increasing time trend was substantially attenuated after adjusting for methodological variation and geographical areas, and regional trends showed considerable fluctuations.
- The updated estimated number of people with dementia in all these areas is 9.5 million, which is higher than the previous estimate (8.4 million).

## Introduction

Dementia, a syndrome of cognitive decline and a major cause of disability in older age, has become a global public health priority in the context of population ageing.^1^^,^^2^ The worldwide epidemiology of dementia has been an important topic, as it provides fundamental information for dementia research, charity lobbying and policy planning.^1^ Since the turn of the millennium, many prevalence studies of dementia have been conducted in low- and middle-income countries, with China being among these.^3^ Estimated prevalence and number of people with dementia in China have been reported in some multicentre prevalence studies^4–6^ and systematic reviews.^7–12^

Although the earlier literature suggests a lower prevalence in China compared with Western Europe and other high-income countries,^1^^,^^7^^,^^13^ systematic reviews including more recent studies have reported higher estimates and indicated a dramatic increase in prevalence over time.^8^^,^^9^^,^^12^ However, these analyses did not fully take into account methodological features of individual studies. Changes in diagnostic criteria and research methods can influence dementia case identification considerably, and therefore these results might not reflect the true prevalence trend in the Chinese older population. In addition, the existing reviews have generally estimated the number of people with dementia based on a single set of pooled prevalence estimates, and have not considered variation within this region.^9^^,^^12^ China, Hong Kong and Taiwan have had very different historical, economic and societal contexts as well as various trajectories of life expectancy and health status. Variations within China, one of the largest countries in the world, have seldom been fully explored.

Our earlier meta-analytical review has covered the prevalence studies of dementia from mainland China, Hong Kong and Taiwan published before April 2012, and identified important methodological factors related to the heterogeneity of prevalence estimates. The findings reveal north-south geographical variation and a fluctuating time trend in dementia prevalence when taking into account methodological factors.^10^^,^^11^ With rising global attention to population ageing and dementia, several new prevalence studies have been conducted in China, Hong Kong and Taiwan and published in the past 5 years. This provides an opportunity to update prevalence data in these areas and review the findings from previous analyses. Building on our earlier reviews,^10^^,^^11^ the analysis here updates the prevalence estimates for mainland China, Hong Kong and Taiwan and investigates whether adding these new data changes the results from the previous analyses.

## Methods

### Literature search and data extraction

This study included three systematic reviews: our earlier review (up to April 2012),^11^*World Alzheimer Report 2015* (January 2011–March 2015)^12^ and an updated search (March 2015–February 2017). The literature searches were conducted to identify prevalence studies of dementia in English (PubMed, Web of Knowledge) and Chinese databases (Chinese National Knowledge Infrastructure (CNKI), WanFang and Airiti Library). The same search strategy and inclusion/exclusion criteria reported in the earlier review^10^ were used to select included studies for *World Alzheimer Report 2015*^12^ and the update search. The PRISMA guidelines^14^ were followed and more detailed information on the search strategy is reported in Supplementary material S1, available as Supplementary data at *IJE* online. Inclusion criteria were: (i) cases were collected by field survey, not based on hospital data; (ii) the study involved population sampling rather than recruiting volunteer participants; (iii) the study reported prevalence in people aged 50 and over; and (iv) dementia case identification was not solely decided by a screening test and specific instruments and criteria were reported. Studies were excluded if they were: (i) duplicate; (ii) irrelevant or with other focuses (such as mild cognitive impairment); (iii) the results of follow-up waves; and (iv) focused on Chinese populations outside mainland China, Hong Kong and Taiwan. A full list of included and excluded studies is provided in Supplementary material S1.

Information on study design (sampling method, one/two-stage investigation), methodological factors (screening tools, diagnostic criteria and instruments), characteristics of population (sample size and response rate, the whole study age range and locations) and results (prevalence of all types of dementia and stratified prevalence by age) was extracted from each study. The results of a recent study in Hong Kong^15^^,^^16^ were obtained from a government document, as the data of dementia prevalence have not been fully published in peer reviewed journals. Data extracted from the new prevalence studies were added to the earlier 76 studies, and study quality was assessed based on sample size, study design, response rate and diagnostic assessment.^12^ More detailed information on characteristics and quality assessment of all included studies is provided in Supplementary material S2, available as Supplementary data at *IJE* online.

### Geographical areas

The provinces and cities in mainland China were categorized into three large geographical areas: north (Beijing, Hebei, Heilongjiang, Henan, Liaoning, Shaanxi, Shandong, Shanxi and Tianjin), central (Anhui, Chongqing, Hubei, Hunan, Jiangsu, Jiangxi, Shanghai, Sichuan and Zhejiang) and south (Fujian, Guangdong, Guangxi, Guizhou and Hainan).^10^ Sine the update search found additional studies from north-western areas, studies from Xinjiang and Gansu were separated and categorized into one group (west). Studies from Taiwan and Hong Kong were combined in one group. One multicentre study^6^ including five study centres (Changchun, Beijing, Zhengzhou, Guiyang and Guangzhou) was categorized in one group with other multicentre studies.

### Time periods

Time period was categorized into six groups on the basis of the initial year of investigation (not publication year): before 1990, 1990–94, 1995–99, 2000–04, 2005–09 and 2010–15. Compared with the previous review, the last period group was further divided into two groups as the new prevalence studies were generally conducted after 2010. For studies that did not report the year of investigation in the paper, the publication year minus 3 years was used as an approximation for the survey date.

### Data analysis

To compare results from the earlier review and this update, the same analytical methods reported in the previous analysis^10^ were used to analyse the data. Prevalence estimates extracted from individual studies were standardized to the Census Population of China 2010.^17^ A random-effect meta-analysis was used to calculate pooled estimates of overall prevalence among all included studies (age 50 or above) as well as stratified prevalence by 5-year age groups, gender, methodological factors, geographical areas and time periods. I-square was used to indicate consistency of results across studies.^18^ An age-standardized meta-regression was conducted to explore whether the variation in prevalence estimates can be related to methodological factors or characteristics of study populations, and to investigate difference across geographical areas and time periods taking into account study design and methodological factors. A univariable model was conducted to identify important methodological factors related to variation in prevalence estimates, and the models for geographical areas and time periods were carried out separately. A multivariable model was fitted including geographical areas, time periods and all important methodological factors identified from the univariable analysis. To investigate trends in geographically defined areas, subgroup analysis was conducted of the 24 studies in Beijing (north) and Shanghai (central), the two areas with the earliest studies in the pre-1990 period group, as well as northern and central areas.

The results of meta-regression modelling were used to estimate the number of people with dementia, taking into account methodological factors. Predicted prevalence by the five areas was estimated from the full model including methodological factors, geographical areas and time periods. These estimates were based on Diagnostic and Statistical Manual of Mental Disorders (DSM)-IV/-IV-R, the relatively new diagnostic criteria for dementia among the included studies, and were calculated for the population aged 60 or above in China and aged 65 or above in Hong Kong and Taiwan, due to difference in life expectancy and age range of the included studies. Age-stratified prevalence was calculated based on regional estimates from meta-regression modelling and the assumption of doubling prevalence with every 5 years, which has been confirmed by worldwide evidence on dementia epidemiology.^1^ The stratified prevalence by 5-year age groups applied to population structures in China, Hong Kong and Taiwan. More detailed information on calculation methods is provided in Supplementary material S3, available as Supplementary data at *IJE* online.

## Results

**Figure 1:**
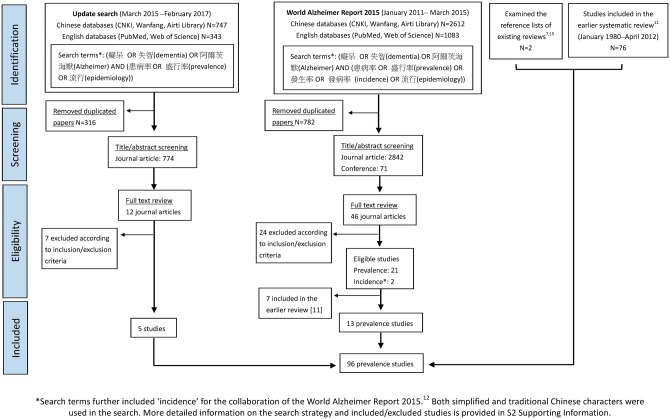
Flow chart of literature search.

The literature search identified 22 studies published between April 2012 and February 2017 (Figure 1). Fifteen were in Chinese and seven in English. Two English papers contained the same information as the Chinese publications.^19^^,^^20^ Information from 20 studies was added to the earlier prevalence database. In total, 96 prevalence studies of dementia (76 from the previous review) were included in this analysis. Among the 20 new studies, 18 were from China and two recent studies were found in Taiwan^30^ and Hong Kong.^15^^,^^16^

**Figure 2:**
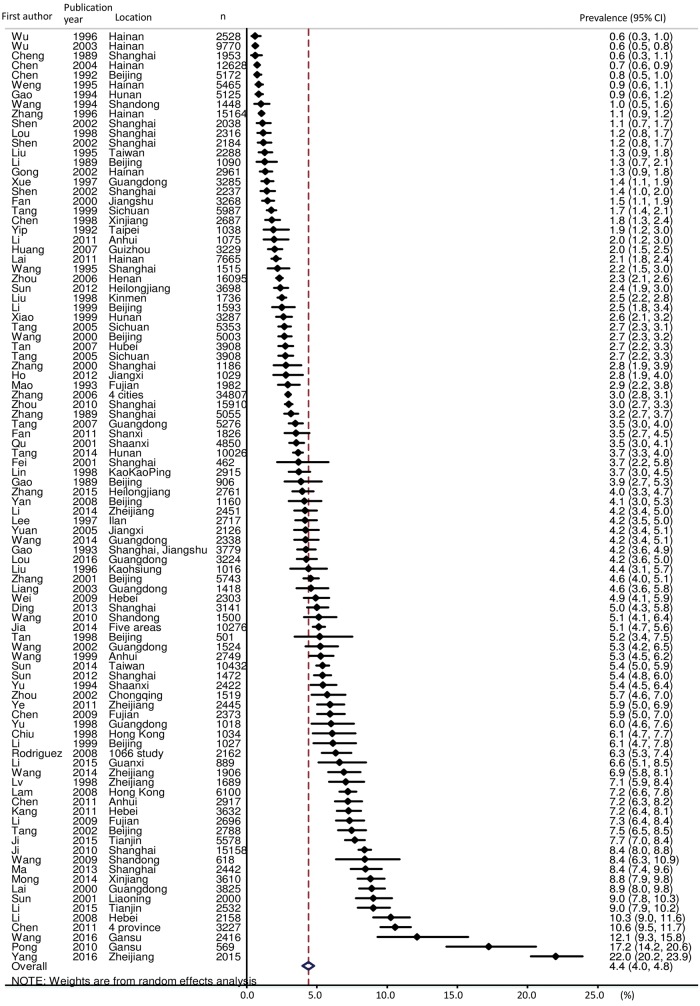
Forest plot of crude prevalence: 96 included studies reporting prevalence in people aged 50 or above.

Among the 96 studies reporting the prevalence of dementia in people aged 50 or above, the pooled estimate was 4.4% [95% confidence interval (CI): 4.4, 4.8] with a range from 0.6% to 22.0% (Figure 2). The heterogeneity was extremely high (I^2^ = 98.6%). The overall estimates were 3.7% (95% CI: 3.2, 4.1) in men and 5.6% (95% CI: 5.0, 6.2) in women. Age-stratified prevalence of dementia was approximately doubling every 5 years of increment of age: 50–54 (0.3%; 95% CI: 0.1, 0.7), 55–59 (0.5%; 95% CI: 0.3, 0.7), 60–64 (1.1%; 95% CI: 0.8, 1.4), 65–69 (2.0%; 95% CI: 1.6, 2.3), 70–74 (3.6%; 95% CI: 3.0, 4.2), 75–79 (5.9%; 95% CI: 5.1, 6.8), 80–84 (10.9%; 95% CI: 9.3, 12.4), 85–89 (18.5%; 95% CI: 14.5, 22.4), 90+ (28.6%; 95% CI: 24.3, 32.9). Six studies did not report age-stratified prevalence and therefore age standardization was applied to 90 surveys. The overall estimate of age-standardized prevalence was 4.5% (95% CI: 4.1, 4.9; I^2^ = 98.4%).

As in the previous review, diagnostic criteria, whole study age range, population size and sampling method remained important in explaining the heterogeneity across individual studies (Model 1, Table 1). Studies using DSM-III/III-R, the International Classification of Diseases (ICD) and the Chinese Classification of Mental Disorders (CCMD) and mixed criteria (pooled estimate: 2.5%, 95% CI: 2.1, 2.9) generally reported lower prevalence than those using DSM-IV/IV-R and other criteria (pooled estimate: 5.7%; 95% CI: 5.0, 6.5). Pooled estimates of prevalence increased with whole study age range. Studies with large sample sizes (5000+) reported lower prevalence than those with sample size less than 5000. Studies conducting cluster-based sampling were likely to report lower prevalence compared with those using other types of sampling methods.

**Table 1:** Meta-regression: the relationships between prevalence estimates, methodological factors, geographical areas and time periods

		Model 1			Model 2			Model 3			Model 4		
		Coeff.	95% CI	*P*	Coeff.	95% CI	*P*	Coeff.	95% CI	*P*	Coeff.	95% CI	*P*
Diagnostic	DSM-III/-III-R (ref.^a^)	–	–	< 0.01	–	–	< 0.01	–	–	< 0.01	–	–	< 0.01
criteria^b^	DSM-IV/-IV-R	3.16	(1.93, 4.40)		2.35	(1.27, 3.44)		2.42	(0.79, 4.05)		2.06	(0.54, 3.58)	
	ICD-10	1.72	(−0.64, 4.09)		0.74	(−1.24, 2.73)		0.57	(−1.86, 2.99)		0.39	(−1.80, 2.58)	
	CCMD	0.69	(−1.67, 3.05)		−0.80	(−2.87, 1.27)		−0.60	(−3.20, 2.00)		−1.20	(−3.51, 1.10)	
	Mixed	2.31	(0.51, 4.10)		1.33	(−0.25, 2.90)		1.99	(0.01, 3.97)		1.54	(−0.25, 3.34)	
	Other	5.30	(3.23, 7.36)		3.29	(1.44, 5.14)		4.04	(1.64, 6.44)		3.25	(1.08, 5.42)	
Whole study	50+	−2.62	(−6.58, 1.33)	< 0.01	−3.41	(−6.58, −0.23)	< 0.01	−0.70	(−4.27, 2.88)	0.01	−2.49	(−5.85, 0.88)	< 0.01
age range	55+	−1.28	(−2.91, 0.35)		−0.97	(−2.29, 0.35)		−1.03	(−2.47, 0.41)		−0.62	(−2.00, 0.76)	
	60+ (ref. ^a^)	–	–		–	–		–	–		–	–	
	65+	1.57	(0.18, 2.97)		1.51	(0.36, 2.67)		1.25	(−0.07, 2.56)		1.45	(0.23, 2.67)	
	70+	3.44	(−0.60, 7.47)		3.79	(0.66, 6.92)		4.21	(0.67, 7.75)		4.81	(1.49, 8.13)	
Sampling	Cluster-based (ref.^a^)	–	–		–	–					–	–	
method	Individual-based	1.96	(0.55, 3.37)	0.02	1.09	(−0.12, 2.30)	0.21	0.98	(−0.20, 2.16)	0.25	1.04	(−0.16, 2.25)	0.23
	Unknown	−0.24	(−3.65, 3.18)		0.31	(−2.24, 2.87)		−0.21	(−3.04, 2.63)		0.38	(−2.20, 2.96)	
Study size	Less than 5000 (ref.^a^)	–	–		–	–					–	–	
	More than 5000	−1.99	(−3.45, −0.54)	0.01	−1.35	(−2.49, −0.20)	0.02	−1.64	(−2.87, −0.45)	< 0.01	−1.29	(−2.43, −0.14)	0.03
Area	North (ref.^a^)	–	–	< 0.01	–	–	< 0.01				–	–	
	Central	−1.56	(−3.03, −0.08)		−0.66	(−1.83, 0.51)					−0.96	(−2.18, 0.27)	< 0.01
	South	−1.53	(−3.27, 0.21)		−1.31	(−2.62, 0.01)					−1.70	(−3.10, −0.31)	
	Hong Kong and Taiwan	−1.40	(−3.54, 0.75)		−2.49	(−4.35, −0.63)					−3.02	(−5.01, −1.04)	
	West	3.93	(0.88, 6.97)		3.57	(0.93, 6.22)					2.76	(0.05, 5.47)	
	Multicentre	0.37	(−2.97, 3.71)		−0.02	(−2.63, 2.59)					−0.22	(−2.87, 2.43)	
Year of	2010–13 (ref.^a^)	–	–	< 0.01				–	–	0.05	–	–	0.08
investigation	2005–09	−0.95	(−2.84, 0.93)					−1.18	(−2.75, 0.40)		−0.86	(−2.35, 0.62)	
	2000–04	−2.33	(−4.40, −0.27)					−1.58	(−3.36, 0.19)		−1.00	(−2.66, 0.67)	
	1995–99	−2.45	(−4.17, −0.72)					−1.82	(−3.42, −0.23)		−1.61	(−3.11, −0.11)	
	1990–94	−3.09	(−5.06, −1.11)					−1.56	(−3.79, 0.67)		−0.42	(−2.69, 1.84)	
	Before 1990	−4.39	(−7.17, −1.62)					−1.91	(−4.49, 0.67)		−2.12	(−4.56, 0.32)	
I^2^					96.1%			96.2%			96.0%		

Coeff, coefficient.

a Ref.: reference group.

b Diagnostic criteria: DSM-III/IV: Diagnostic and Statistical Manual of Mental Disorder Third/Fourth Edition; ICD-9/10: International Classification of Diseases 9th/10^th^ editions; CCMD: Chinese Classification of Mental Disease; Mixed: multiple diagnostic criteria including DSM, ICD or CCMD; Other: 10/66 diagnosis algorithm, Geriatric Mental Status and the algorithm of the Automatic Geriatric Examination for Computer Assisting Taxonomy (GMS-AGECAT), National Institute on Aging–Alzheimer's Association guidelines (NIA-AA).

*P*: *P*-value of F-test, except year of investigation, which reports *P*-value of test for trend; Model 1: univariable model; Model 2: adjusted model including area and methodological factors; Model 3: adjusted model including year of investigation and methodological factors, Model 4: adjusted model including methodological factors, year of investigation and geographical area.

I^2^: I-square of meta-regression; indicator of heterogeneity.

The unadjusted pooled estimate for northern China (5.4%; 95% CI: 4.3, 6.4) was higher than for central China (3.8%; 95% CI: 3.1, 4.4) and south China (3.7%; 95% CI: 3.0, 4.4) but was lower than for west China (9.6%; 95% CI: 4.5, 14.8). Pooled prevalence in Hong Kong and Taiwan was 4.0% (95% CI: 2.7, 5.4). The prevalence of dementia varied across geographical areas after adjusting for study design, methodological factors and year of investigation (Table 1). The absolute difference from northern areas of China was about 1% in central areas (−1.0; 95% CI: −2.2, 0.3), 2% in south areas (−1.7; 95% CI: −3.1, −0.3), 3% in Hong Kong and Taiwan (−3.0; 95% CI: −5.0, −1.0) and 3% in west areas of China (2.8%; 95% CI: 0.1, 5.5).

**Figure 3:**
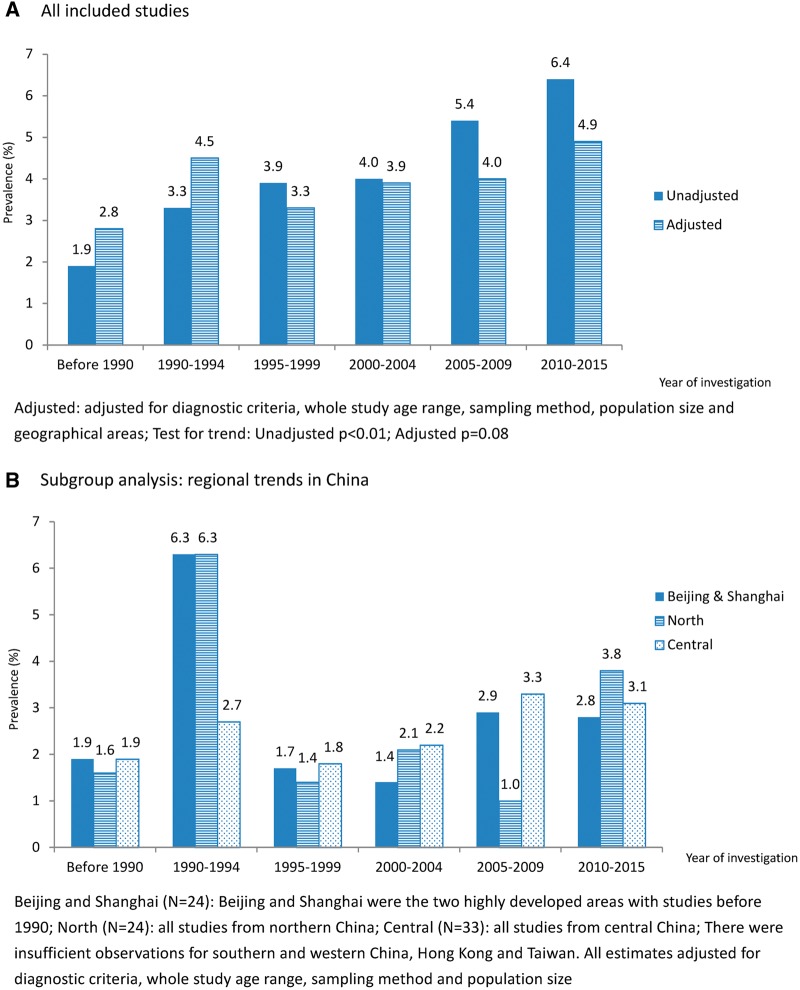
Prevalence estimates in the population aged 60 or above across time periods.

Crude prevalence increased from 1.9% (95% CI: 1.0, 2.9) before 1990 to 6.4% (95% CI: 5.2, 7.7) in 2010–15, with a clear increasing trend. After adjusting for methodological factors and geographical areas, the apparent increasing trend was attenuated (Figure 3A). Although the adjusted estimate in 2010–15 (4.9%; 95% CI: 2.8, 7.0) was nearly twice as high as the prevalence reported from five studies before 1990 (2.8%; 95% CI: 0.4, 5.2), the variation in dementia prevalence after 1990 was unclear and regional trends revealed considerable fluctuation across the time periods (Figure 3B). In particular, trends in 24 studies from Beijing and Shanghai, the only two areas with studies before 1990, showed an even dramatic fluctuation after adjusting for methodological factors.

Based on the results of DSM-IV/IV-R, Table 2 reports estimated numbers of people with dementia by geographical areas, which show substantial difference in meta-regression modelling. In China, the estimated number of people with dementia was 9.5 million in those aged 60 or over (5.3%, 95% CI: 4.3, 6.3) and 3.5 million of these were in northern China (5.5%; 95% CI: 4.3, 6.7). New estimates for people aged 65 or above were 0.07 million in Hong Kong (7.2%; 95% CI: 5.3, 9.1) and 0.15 million in Taiwan (6.0%; 95% CI: 4.1, 7.9).

**Table 2:** Estimated numbers of people with dementia in mainland China, Hong Kong and Taiwan (millions) based on DSM-IV/IV-R criteria

Area		Older population^a^	Number of people with dementia (millions)	Prevalence (%, 95% CI)
China	North	63.9	3.52	5.5 (4.3, 6.7)
(age 60+)^a^	Central	73.3	3.79	5.2 (4.0, 6.4)
	South	30.9	1.48	4.8 (3.5, 6.1)
	West	9.5	0.69	7.2 (4.6, 9.8)
	Total	177.6	9.48	5.3 (4.3, 6.3)
Hong Kong (age 65+)^a^		0.9	0.07	7.2 (5.3, 9.1)
Taiwan (age 65+)^a^		2.5	0.15	6.0 (4.1, 7.9)
Total		181.0	9.70	5.3 (4.3, 6.3)

a The estimation for Hong Kong and Taiwan focused on the population aged 65 or over as most studies in these two areas recruited participants aged 65 or over.

## Discussion

This updated review has identified 20 prevalence studies in addition to the 76 studies included in the earlier review, and confirmed geographical variation and time trends reported in the previous analyses.^13^^,^^14^ The prevalence of dementia decreases from northern, central and southern China to the lowest in Hong Kong and Taiwan, but a particularly high estimate was found in western China. The apparent increasing prevalence across time is attenuated once adjusted for methodological factors and geographical areas. Regional patterns over time reveal considerable fluctuations. The current best estimate for number of people with dementia in this region as a whole is 9.5 million (5.3%; 95% CI: 4.3, 6.3) in the population aged 60 or above.

Compared with other existing systematic reviews, this analysis investigated geographical variation and time trends taking important methodological factors and characteristics of study population into account, and used regional prevalence estimates and population data to model number of people with dementia by different areas. To examine the potential interaction of geographies and time, this review further explored regional trends in Beijing and Shanghai since the late 1980s.

There are some limitations in this review. The three literature searches were conducted at different time points, due to limited research funding and resources. Although this might introduce bias, the updated searches included a short overlapping period from the earlier searches in order to ensure that the same studies were identified. Results of literature searches were also compared with the reference lists of existing systematic reviews. The study protocol was not registered or published before the review being conducted, but this update generally followed the study methods and procedures used in our earlier studies. It is possible that unpublished data or local investigations might exist for less developed areas but are not available in the public domain. Although some new investigations have been conducted in north-western provinces, most studies are concentrated in Shanghai and relatively wealthy areas. The current estimates are still mainly based on the studies existing in highly developed areas and, from the indicators of regional variation, these might not fully represent substantial variation across China. Although the analysis investigated potential sources of heterogeneity, considerable inconsistency of prevalence estimates across studies could not be fully explained by methodological variation. It is possible that some unmeasured factors and characteristics of study population, such as mortality, might influence prevalence estimates in the study population, but such information could not be extracted from the publications. The number of people with dementia was calculated based on the modelling results, and therefore estimates are sensitive to small differences in regression coefficients. Although changes in prevalence did not achieve statistical significance, the numbers calculated using the time point estimate will still vary substantially because of the sheer size of the Chinese older population.

The study quality was not related to variation in prevalence estimates and did not considerably change over time. However, most two-stage studies did not include a sample of screen negatives and appropriate weights. The quality of reporting also varied across individual studies. For example, although two-thirds of studies had high response rates (> 80%), 22% did not report such information and selection bias in these studies was unknown. Some short reports did not provide detailed information on research methods or study populations. Such lack of detail may lead to unexplained heterogeneity in the meta-analysis. In addition, a relatively small number of studies (9%) used comprehensive diagnostic assessment, including multi-domain cognitive tests, disability assessment and informant and clinical interviews. Implementation of dementia diagnosis might be compromised due to incomplete information on cognitive and functional status.

The 20 new studies contributed a quarter of the total database and continue to highlight potential variation in cognitive health within East Asia. Our earlier review and this update suggest similar findings of geographical variation: prevalence estimates were higher in the north, with lower estimates further south after adjustment for methodological factors. In addition, studies from western China appear to provide distinct estimates compared with the north in this new analysis, and showed a particularly high pooled estimate. This might indicate a complicated and prolonged influence of societal contexts on individual life experiences, behaviours and health conditions, which may affect health and cognition in later life. Regional differences in life course exposures such as education, smoking, nutrition and diet,^31^ as well as potential environmental risk factors such as sunshine and Vitamin D intake,^32^ air pollution^33^ and health services,^34^ may play a part in general health and subsequent risk of dementia and provide a possible explanation of geographical variation. In particular, Uyghur and other ethnic minorities in western areas have very different lifestyle, culture and environment from the Han Chinese and generally experience high levels of deprivation.^35^ Variation in education, general health and life experiences between Uyghur and Han might contribute to differences in cognitive health at older ages. Urban and rural differences have been reported in a recent multicentre study^6^ but the analysis here could not explore specific estimates for urban-rural areas due to different definitions of urban and rural settings across studies. Improving reporting on geographical locality characteristics would enable this to be explored further.

The new studies have provided more data on prevalence estimates in the past 5 years. The unadjusted pooled prevalence has increased over time with the highest in the most recent period group (2010–15), but this difference can be largely attributed to changes in diagnostic criteria and study methods as well as geographical variation within China. The findings confirm an increasingly recognized phenomenon of the major influence of diagnostic methods on prevalence estimates.^36^ Further adjustment for geographical areas and subgroup analyses on regional trends also considerably attenuated increasing prevalence across time. Although the adjusted estimate in 2010–15 was twice as high as the one for the oldest group (before 1990), the estimate from the pre-1990 period is highly atypical, as these early studies only focused on relatively small areas in metropolitan cities (Beijing and Shanghai). The research context of these old investigations is therefore very different from that of more recent studies. In particular, the second edition of the Chinese Classification of Mental Disorders (CCMD-2) was published in 1989.^37^ Development of psychiatric knowledge and the adaptation of new criteria change case identification and prevalence estimates across time. Although several existing reviews have suggested that the prevalence of dementia might have increased across time, given the rising incidence of chronic conditions such as diabetes, vascular diseases and metabolic syndrome,^12^^,^^38^ the scale of increase cannot be determined unless the effect of different diagnostic criteria and methodologies can be accounted for first. What is unclear in all the changes to diagnostic criteria over the time periods is how differently each set of diagnostic criteria predicts natural history and, indeed, whether the most recent changes lead to greater misclassification (i.e. more false positives).

Despite marginal changes in prevalence estimates, the new estimated number of people (9.5 million) is one million higher than the previous estimate (8.4 million). In the *World Alzheimer Report 2015*, the estimate for East Asia was 9.8 million based on recent studies in China (published after 2005).^12^ Although these differences reflect different statistical aspects of estimation and varying sources of standard population data, small changes in prevalence estimates could indicate enormous impact on health and social care systems and the whole society. The changing meaning of dementia diagnoses and measurement modalities needs close attention, as an increase of 1 million people at the very mild end of the spectrum, which may include false positives or people with low likelihood of progression, has very different implications for society compared with an increase of 1 million moderately to severely affected people.

Although population ageing, changes in lifestyle and rise in chronic diseases might increase the risk of dementia in older populations,^31^ recent epidemiological studies in high-income countries have reported stable or reduced prevalence of dementia over the past 2 decades.^39^ Possible explanation of these decreasing trends has been related to the improvement of education, living conditions and lifestyle and reduction in chronic conditions.^40^ Although overall prevalence trends in mainland China, Hong Kong and Taiwan are uncertain due to the substantial impact of methodological variation, geographical variation within this region might provide an opportunity to investigate these hypotheses. Differences in economic and societal development across China may be potential contexts for natural experiment research.

High quality primary research is needed in this region in order to provide robust prevalence estimates and evidence for policy planning. In recent years, international organizations and civil societies have focused on promoting national plans for dementia.^41^ In addition to these dementia-specific policies, public health policy planning needs to adopt a comprehensive approach to improve general health in populations and to address determinants of cognitive health across the life course, in order to inform prevention or risk reduction strategies.^40^

## Supplementary Data

Supplementary data are available at *IJE* online.

## Funding

This work was partially supported by the World Alzheimer Report 2015, Alzheimer's Disease International (http://www.alz.co.uk/), for the updated literature search of January 2011-March 2015. AMP was supported by the UK Medical Research Council (MR/K021907/1). The funders had no role in study design, data collection or analysis, decision to publish or preparation of the manuscript.

## Supplementary Material

Supplementary Data
