# Supplementary material for: Prevalence of dementia in mainland China, Hong Kong and Taiwan: an updated systematic review and meta-analysis
Source: Int J Epidemiol. 2018 Feb 12;47(3):709–19. doi: 10.1093/ije/dyy007 (PMC6005065; doi:10.1093/ije/dyy007)
Supplement: Supplementary Data [file dyy007_ije-2017-03-0282-file006.pdf]

Prevalence of dementia in mainland China, Hong Kong, and Taiwan: an updated systematic review and meta-analysis

Supplementary material

S1. Protocol, search strategy, included and excluded studies

S2. Characteristics and quality of included studies (N=96)

S3. Estimation on number of people with dementia

S4. PRISMA checklist

Appendix S1. Protocol, search strategy, included and excluded studies

1. Systematic review protocol

Background

Dementia has become a major public health concern across the globe.[1] The worldwide epidemiology of dementia has been an important topic as it provides fundamental information for research and policy planning. Compared to other low and middle income countries, China has a comparatively rich body of literature on prevalence studies of dementia.[2] Although there are several existing systematic reviews of prevalence of dementia for mainland China, Hong Kong and Taiwan (latest search until April 2012),[2-4] the recent reporting of contemporary provides a need for these to be updated.

Aim

The aim of this study is to update the prevalence estimates of dementia in mainland China, Hong Kong and Taiwan and examine geographical variation and time trends incorporating the new studies. The objectives of this review include:

(1) To identify prevalence studies of dementia in mainland China, Hong Kong and Taiwan published after April 2012

(2) To estimate the prevalence and numbers of people with dementia using new dataset

(3) To examine variations across geographical areas and time periods with the adjustment for methodological factors

Method

Search Strategy

The same search strategy and inclusion/exclusion criteria reported in the earlier reviews will be used to select included studies.[4] An electronic search of English and Chinese databases: PubMed, Web of Science, Chinese

National Knowledge Infrastructure (CNKI), Wanfang and Airti Library will be conducted to identify publications on prevalence studies. The search strategy has been developed by leading authors of previous systematic reviews[2-4] and the reference lists of existing reviews[5-7] will be checked to identify unpublished studies or reports. The following table shows the English and Chinese search terms employed in the World Alzheimer Report 2015 (January 2011–March 2015) and the updated search (March 2015–February 2017):

The search strategy used in the World Alzheimer Report 2015

English keywords	Chinese keywords[1.]
Database: PubMed, Web of Knowledge	Database: CNKI, WanFang and Airti Library
Publication year: January 2011~March 2015	Publication year: January 2011~March 2015
Dementia OR Alzheimer*	癡呆、失智、阿爾茨海默
AND	
Prevalence OR Incidence[3] OR Epidemiology	患病率、盛行率、發生率、發病率、流行
AND	
China OR Chinese OR Taiwan OR Taiwanese	-[2]

The search strategy used in the updated review

English keywords	Chinese keywords[1.]
Database: PubMed, Web of Knowledge	Database: CNKI, WanFang and Airti Library
Publication year: March 2015~Feburary 2017	Publication year: March 2015~Feburary 2017
Dementia OR Alzheimer*	癡呆、失智、阿爾茨海默
AND	
Prevalence OR Epidemiology	患病率、盛行率、流行
AND	
China OR Chinese OR Taiwan OR Taiwanese	-[2]

[1.] Both traditional and simplified Chinese characters were used in the literature search.

[2.] These terms were not used in the search in Chinese databases.

[3.] Search terms further included ‘incidence’ for the collaboration of the World Alzheimer Report 2015

Title screening and full text review will be conducted by two reviewers based on the following inclusion and exclusion criteria:

Inclusion criteria

(1) Cases were collected by field survey, not based on hospital data

(2) The study involved population sampling rather than recruiting volunteer participants

(3) The study reported prevalence in the people aged 50 and over

(4) Dementia case identification was not solely decided by a screening test and that specific instruments and criteria were reported

Exclusion criteria

(1) Duplicate studies and follow-up studies of fixed population

(2) Irrelevant studies or with other focuses (such as Alzheimer's subtype or mild cognitive impairment)

(3) The results of follow-up waves

(4) The study population which investigated the prevalence of the Chinese population but outside of mainland China, Hong Kong and Taiwan

Data extraction

The following information will be extracted from the included studies:

(1) Study design: methods of screening, diagnosis and confirmation, interviewers and sampling method;

(2) Participants: sample size and response rate, characteristics of participants, such as age group, study location, urban or rural area;

(3) Dementia identification: screening tools, diagnostic criteria and instruments;

(4) Results: overall prevalence of all type dementia and stratified prevalence by age, gender and educational level.

Data analysis

The same analytical methods reported in the previous analysis[4] will be used to analyse the up to date prevalence data. To adjust for the effect of age, prevalence estimates from individual studies will be standardised to the

Census Population of China 2010.[8] A random-effect meta-analysis will be used to calculate the overall and regional pooled estimate of dementia prevalence. Meta-regression will be conducted to test whether the variation in prevalence estimates can be related to methodological factors or characteristics of study populations and to investigate difference across geographical areas and time periods taking into account study design and methodological factors. The results of meta-regression modelling will be applied to population structures by different areas and used to estimate the numbers of people with dementia.

References

1 World Health Organization. *Dementia: a public health priority*. Geneva: World Health Organization, 2012.

2 Alzheimer's Disease International. *World Alzheimer Report 2015: the global impact of dementia: an analysis of prevalence, incidence, cost and trends*. London: Alzheimer's Disease International, 2015.

3 Chan KY, Wang W, Wu JJ *et al*. Epidemiology of Alzheimer's disease and other forms of dementia in China, 1990–2010: a systematic review and analysis. *Lancet* 2013;**381**:2016-23.

4 Wu Y-T, Lee H-y, Norton S *et al*. Prevalence studies of dementia in mainland China, Hong Kong and Taiwan: a systematic review and meta-analysis. *PLoS One* 2013;**8**:e66252.

5 Zhang Y, Xu Y, Nie H *et al*. Prevalence of dementia and major dementia subtypes in the Chinese populations: A meta-analysis of dementia prevalence surveys, 1980-2010. *J Clin Neurosci* 2012;**19**:1333-7.

6 Yu R, Chau PH, McGhee SM *et al*. Trends in prevalence and mortality of dementia in elderly Hong Kong population: projections, disease burden, and implications for long-term care. *Int J Alzheimers Dis* 2012;**2012**:6.

7 Liu BY, Wang JL, Xiao YZ. Prevalence of senile dementia in people aged ≥60 years in China: a meta-analysis. *Zhonghua Liu Xing Bing Xue Za Zhi* 2016;**37**:1541-1545.

8 National Bureau of Statistics of China. *China Statistical Yearbook 2010*. Beijing: National Bureau of Statistics of China, 2011.

2. Included studies (new studies marked in red)

[1] Ye GC, Chen FR, Shen KZ. Dementia in people over 60 years old and related risk factors. *Chinese Rural Health Service Administration* 2011;**31**:152-154.

[2] Li L, Li F, Ma Y *et al*. Prevalence of dementia among the elderly in rural community of Anhui. *Chin J Dis Control Prev* 2011;**15**:292-294.

[3] Zhao QZ, Ding D, Guo Q, Hong Z. Prevalence, mortality, and predictive factors on survival of dementia in Shanghai, China. *Alzheimer Dis Assoc Disord* 2010;**24**:151-158.

[4] Ji M, Yao XW, Lu J. A cross-sectional study of senile dementia in Shanghai. *Chin J Rehabil Theory Pract* 2010;**16**:513-515.

[5] Wang F, Xu, P, Zhu, S. Survey on the awareness rate and prevalence of Alzheimer's disease in the urban area of Zaozhuang city. *Chinese Journal of Practical Nervous Diseases* 2010;**13**:9-11

[6] Chen B, Luo WW, Chen LL *et al*. Condition of senile dementia and analysis of its dangerous social-psychological factors. *Fujian Med J* 2009;**31**:133-136.

[7] Li H, Zhang H, Hung H, Wang YZ, Hung HL. Prevalence of dementia among rural elderly in Gushan township, Fuzhou. *Chin J Epidemiol* 2009;**30**:772-775.

[8] Wang H, Zhang YQ, Chen XR. Epidemiological survey of senile dementia among elderly people in communities of Tai'an. Chin J Public Health 2009;**25**:899-900.

[9] Rodriguez J, Ferri C, Acosta D *et al*. Prevalence of dementia in Latin America, India, and China: a population-based cross-sectional survey. *Lancet* 2008;**372**:464-474.

[10] Wen H, Zhang H, Xu L. Epidemiological survey of dementia in Boading city. *Modern Preventive Medicine* 2008;**35**:847-848.

[11] Yan F, Li SR, Huang YQ. Longitudinal study on dementia in an urban community of Beijing city in two decades. *Chinese Mental Health Journal* 2008;**22**:110-113.

[12] Huang W, Yang X, Yang JY. Investigation on prevalence of dementia among elderly in urban communities of Guiyang city. *Chin J Public Health* 2007;**23**:983-984.

[13] Tan J. The survey of prevalence of dementia in the ethnical district of Hubei. *Basic Medicine Forum* 2007;**11**:703-705.

[14] Tang M, Ma C, Huang XM *et al*. The prevalence of dementia in urban and rural areas in Guangzhou. *Chin J Nerv Ment Dis* 2007;**33**:340-344.

[15] Zhang Z, Zahner G, Roman G *et al*. Socio-Demographic Variation of Dementia Subtypes in China: Methodology and Results of a Prevalence Study in Beijing, Chengdu, Shanghai, and Xian. *Neuroepidemiology* 2006;**27**:177-187.

[16] Zhao D, Wu CS, Qi H *et al*. Prevalence of dementia in rural China: impact of age, gender and education. *Acta Neurol Scand* 2006;**114**:273–280

***[17]** Tang M, Liu X, Lu J. Comparison of prevalence of dementia between in 1997 and 2000 surveys in the urban and rural community in Chengdu. *Chin J Psychiatry* 2005;**38**:170-173.

[18] Wang D, Jia X. The investigation of dementia and related risk factors. *Chin J Public Health* 2002;**18**:1498-1499.

[19] Tang Z, Meng C, Dong HQ *et al*. An epidemiological study in urban and rural areas in Beijing. *Chin J*

Gerontology 2002;**22**:224-246.
*[20] Shen C, Li HM, Guo LN. A follow-up study on prevalence of senile dementia in rural area. *Shangdong Act Psychiatry* 2002;**15**:187-189.
[21] Zhou K, Qi J, He Y, Zhang SH, Li G. A cross-sectional study of senile dementia in Liang Lukou street of Yuzhong county of Chongqing. *Chin J Geriatr* 2002;**21**:433-435.
[22] Zhang Z, Wei J., Hong X. Prevalence of dementia and major subtypes in urban and rural communities of Beijing. *Chin J Neurol* 2001;**34**:199-203.
[23] Qu Q, Qiao J, Yang JB. Study of the prevalence senile dementia among elderly people in Xian, China. *Chin J Geriatr* 2001;**20**:283-286.
[24] Sun Y, Zhou M, Wu C *et al*. The investigation of dementia prevalence in Dalian. *Chin J Psychiatry* 2001;**34**:26.
[25] Fei W, Cheng L, Wang YM. An analysis on senile dementia prevalence at a community in Luwan district, Shanghai. *Sichuan Mental Health* 2001;**14**:31-32.
[26] Lai S, Wen ZH, Liang WX. Prevalence of dementia in an urban population aged ≥75 years in Guangzhou. *Chin J Geriatr* 2000;**19**:450-455.
[27] Wang W, Wu SP, Cheng XM *et al*. Prevalence of Alzheimer's Disease and Other Dementing Disorders in an Urban community of Beijing, China. *Neuroepidemiology* 2000;**19**:194-200.
[28] Zhang Z. An epidemiological distribution on dementia in Baoshan district, Shanghai. *J Health Psychology* 2000;**8**:536-538.
[29] Fan J, Yan JL, Chen ZH. An epidemiological report of senile dementia in Nanjing area. *J Clin Psychol Med* 2000;**10**:137-138.
[30] Li S, Chen CH, Zhang WX *et al*. The Prevalence of Dementia and Depression of the Elderly in the Urban Community in Beijing. *Chinese Mental Health Journal* 1999;**13**:266-268.
[31] Li Z, Dong ZX, Lv SX. The situation of prevalence of dementia in Dongcheng District, Beijing. *Chin J Geriatr* 1999;**19**:3-4
[32] Xiao Z, Li OX, Liao EY. (1999) An epidemiological study on dementia in Furong district, Changsha. Chin J Clin psychology 7: 221-223.
[33] Wang T, Sui JZ, Wei XB. The prevalence of senile dementia in urban and rural areas of Anhui province. *Occup Health* 1999;**15**:43-45.
[34] Tang M, Guo YB, Xiang MZ. Epidemiology of senile dementia and Alzheimer disease in the rural area. *J Clin Psychol Med* 1999;**19**:20-22.
[35] Chiu HFK, Lam LCW, Leung T *et al*. Prevalence of dementia in Chinese elderly in Hong Kong. *Neurology* 1998;**50**:1002-1009.
[36] Lou K, Char Y, Hu D *et al*. The investigation of senile dementia in Chinpu county. *Shanghai Journal of Preventive Medicine* 1998;**10**:187-189.
[37] Lin R, Lai CL, Tai CT *et al*. Prevalence and subtypes of dementia in southern Taiwan: Impact of age, sex, education, and urbanization. *J Neurol Sci* 1998;**160**:67-75.
[38] Yu J, Fei Z, Fang X *et al*. The survey of prevalence of senile dementia in the rural area of Guangdong. *Chin J Psychiatry* 1998;**24**:30-33.

[39] Wang J, Yu JH, Pan SZ, Ni BL. Investigation of senile dementia in Qingdao. *Psychiatry Clin Neurosci* 1998;**52**:S295–S296.
[40] Lv S, Yu HM, Chen YH, Hu BY. Epidemiological survey of senile dementia in Dinghai District, Zhoushan City residents. *Chinese Mental Health Journal* 1998;**31**:225-227.
[41] Chen Z, Zhang L., Lai YX. The epidemiology of vascular dementia and Alzheimer's dementia in Shihezi, Xinjiang, China. *Chin J Neuroimmunol Neurol* 1998;**5**:44-48.
[42] Liu H, Fuh JL, Wang SJ *et al*. Prevalence and subtypes of dementia in a rural Chinese population. *Alzheimer Dis Assoc Disord* 1998;**12**:137-144.
[43] Xue G, Shao YC, Zhu GZ. An epidemiological study on dementia in Guangdong province. *J Pract Med (Chin)* 1997;**13**:371-372.
[44] Wu C, Wang X, Lou H *et al*. Epidemiological survey of senile dementia in Hainan. *Sichuan Mental Health* 1996;**9**:1–2
[45] Zhang H, Wen G, Liao X *et al*. Epidemiological investigation of dementia in Hainan. *Hainan Medicine* 1996;**3**:207-208.
[46] Liu C, Lin RT, Chen YF *et al*. Prevalence of dementia in an urban area in Taiwan. *J Formos Med Assoc* 1996;**95**:762-768.
[47] Wang D, Bu SM. A survey of prevalence of dementia in Shanghai. *Archives of psychiatry* 1995;**7**:76–78
[48] Wen G, Zhang H, Huang Y, Liao X. Epidemiological survey of senile dementia in Haiko city. *Practical Geriatrics* 1995;**9**:186–187
[49] Gao Z, Sheng X, Shang F. Epidemiological survey of senile dementia in Changsha, Hunan. *Hunan Medical Journal* 1994;**11**:195-196.
[50] Mao R, Zhang RR, Ni YX. Prevalence of senile dementia in urban and rural areas of Longyan. *Chin J Gerontol* 1993;**13**:226-228.
[51] Gao Z, Liu FK, Fan YS. A study of morbidity rate of senile dementia among aged in urban and rural areas. *Chinese Mental Health Journal* 1993;**26**:209-212.
[52] Li G, Shen YC, Chen YW, Zhao SR, Li M. An epidemiological survey of age-related dementia in an urban area of Beijing. *Acta Psychiatr Scand* 1989;**79**:557-563.
[53] Zhang M, Qu K, Ren F *et al*. The primary analysis of prevalence of dementia and Alzheimer's disease. *Shanghai Archives of Psychiatry* 1989;**7**:153-159.
[54] Gao S, Zhang LP. A survey of 906 cases of age-related dementia. *Chin J Geriatr* 1989;**2**:75-79.
***[55]** Chen R, Ma Y, Wilson K *et al*. A multicentre community-based study of dementia cases and subcases in older people in China — the GMS-AGECAT prevalence and socio-economic correlates. *Int J Geriatr Psychiatry* 2012;**27**:692-702.
[56] Fan Q, Zheng JZ. Investigation of prevalence of Alzheimer and its risk factors. *Modern Preventive Medicine* 2011;**38**:3029-3033.
[57] Kang M, Gao YM, Huo HQ. Prevalence of Alzheimer's disease and its related factors among elderly people in Hebei province. *Chin J Public Health* 2011;**27**:1123-1125.
[58] Chen C, Shen Y, Li S *et al*. Epidemiological investigation in the western district of Beijing. *China Journal of Health Psychology* 1992;**6**:49-52.

[59] Yip P, Shyu YI, Liu SI *et al*. An epidemiological survey of dementia among elderly in an urban district of Taipei. *Acta Neurol Sin* 1992;**1**:347-354.
[60] Lee C, Chang SF, Su CL, Chen ZY, Chen RC. Neuroepidemiological survey in Ilan, Taiwan (NESIT): (3) An epidemiological survey of dementia in a rural area. *Acta Neurol Taiwan* 1997;**6**:27-35.
[61] Yu X, Zhang CF, Guo MX, Sun YZ. An epidemiological survey of dementia in the elderly in Shaanxi. *Proceedings of the Dementia International Symposium, Hainan province* 1994:1-6.
[62] Sun Z, Cui GC, Han YF, Wei HL. Prevalence of Alzheimer's disease among residents of Fulaerji district of Qigihar city, Heilongjiang province. *Chin J Public Health* 2012;**28**:151-153.
[63] Pong X, Li HC, Zhao Q. The Investigation of Community Elder' Senile dementia in Lanzhou. *Medicine and Society* 2010;**23**:58-59.
[64] Li K, Jiang QP, Cui LJ. Epidemiological Investigation of Alzheimer Disease in the Urban and Rural Areas in Hebei Province. *China Journal of Health Psychology* 2008;**16**:1251-1252.
[65] Yuan Y, Wan AL, Cheng JY *et al*. Epidemiological report of senile dementia in Nanchang city. *Chin J Neuromed* 2005;**4**:65-67.
[66] Liang L, Lin QM, Huang Z. Epidemiological survey of senile dementia among elderly people in community. *Modern Preventive Medicine* 2003;**30**:515-516.
[67] Wu C, Huang JD, Chen WX. Epidemiological investigation of patients with Alzheimer's dementia in Urban area in Hainan Province. *China Tropical Medicine* 2003;**13**:1056-1076.
[68] Tang X, Tian DZ, Liu LS. An epidemiological study of dementia of the aged Hui and Han residents in Niujie area of Beijing. *Chin J Geriatr* 1998;**17**:307-310.
[69] Chen W, Wu CD, Huang JD. Epidemiological survey of Alzheimer's Disease and cerebrovascular dementiain in Hainan. *China Tropical Medicine* 2004;**14**:4016-4018.
[70] Cheng Z, Wang Z, Zhu H *et al*. The investigation of intelligence of 1953 elder people in Shanghai. *Chin J Geriatr* 1989;**9**:204-208.
[71] Lai M, Zhang H, Yi X *et al*. Epidemiological investigation of Alzheimer's disease in Hainan. *Chin J Geriatr* 2011;**31**:4016-4018.
[72] Liu H, Lin KN, Teng EL *et al*. Prevalence and subtypes of dementia in Taiwan: a community survey of 5297 individuals. *J Am Geriatr Soc* 1995;**43**:144-149.
[73] Ho S. Dementia and risk factors in rural communities. *Journal of Jiujiang University (natural sciences)* 2012;**1**:29-32
[74] Sun H, Wu Y, Ye F *et al*. Prevalence of dementia among elderly people in Sheshan town, Songjiang district, Shanghai. *Journal of Internal Medicine Concepts & Practice* 2012;**7**:91-95
[75] Ding D, Zhao Q, Guo Q *et al*. Prevalence survey of dementia among elderly in a urban community in Shanghai. *Chin J Clin Neurosci* 2013;**21**:19-25
[76] Ma Y, Chiang Z, Wang J *et al*. Dementia and subtypes in people aged 65 and over in Shanghai. *Chinese Journal of Gerontology* 2013;**6**:1365-1366
[77] Wang J, Lv B, Ding Y, Ding S. An investigation of dementia in Zhoushan district. *Clinical Education of General Practice* 2014;**12**:213-215
[78] Wang T, Zhen Y, Du W, He X, Chen Z, Guo Q. Alzheimer disease and its influencing factors in urban area

of Dongguan. *International Medicine and Health Guidance News*. 2014;**20**:591-2
[79] Li Q. Analyze and Study the Regional Infection Situation of Alzheimer's Disease. *Journal of Zhejiang Chinese Medical University* 2014;**38**:654-656
[80] Mong X, Liu T, Liu Y *et al*. Survey of the incidence of elderly dementia of Kazak ethnic group Xinjiang Uygur Autonomous Region. *Chin J Neurol* 2014;**47**:491-495
[81] Tang J, Zhou W, Chiang H *et al*. Epidemiological survey and analysis about senile dementia among persons ≥55 years old living in Changsha area. *Journal of Neuroscience and Mental Health* 2014;**14**:164-166
[82] Li H, Hu C, Wei D *et al*. Prevalence of dementia among elderly people in Santang community, Nanning city. *Chinese Journal of Geriatric Care* 2015;**13**:29-31
[83] Sun Y, Lee H-J, Yang S-C *et al*. A Nationwide Survey of Mild Cognitive Impairment and Dementia, Including Very Mild Dementia, in Taiwan. *PLoS One* 2014;**9**:e100303.
[84] Ji Y, Shi Z, Zhang Y *et al*. Prevalence of Dementia and Main Subtypes in Rural Northern China. *Dement Geriatr Cogn Disord* 2015;**39**:294-302
[85] Jia J, Wang F, Wei C *et al*. The prevalence of dementia in urban and rural areas of China. *Alzheimers Dement* 2014;**10**:1-9.
[86] Hong Kong SAR government. *Elderly Commission: Prevalence of dementia in Hong Kong*. Hong Kong: Hong Kong SAR government, 2006. Available: www.elderlycommission.gov.hk/en/download/meeting/Study%20on%20Prevalence%20of%20dementia.pdf
[87] Wang G, Pei G, Xie R *et al*. Prevalence of dementia in the people aged 60 and older of Tianshui. *Int J Psychiatry* 2016;**3**:389-392
[88] Lou Y, Song X, Shao L, Wu X, Zheng R, Zheng S. Investigation of the elderly dementia patients' prevalence and current situation of life quality in Jiangmen as well as countermeasures. *Medical Journal of Chinese People's Health* 2016;**28**:59-61
[89] Zhang DH. Epidemiological survey of senile dementia in community and its feasible countermeasures. *Journal of Clinical Medical Literature* 2015;**2**:507-510.
[90] Yang L, Jin X, Yan J *et al*. Prevalence of dementia, cognitive status and associated risk factors among elderly of Zhejiang province, China in 2014. *Age Ageing* 2016;**45**:707-711
[91] Li C, Yang L, Zhao L *et al*. The prevalence of senile dementia of aged serving by Beichen community hospital in Tianjin. *Prac Prev Med* 2015;**22**:305-307
[92] Gong JB, He ZY, Li DF *et al*. Epidemiological investigation of senile dementia in Danzhou of Hainan province. *Hainan Med* 2002;**13**:52-53

*Tang et al., (2005) and Chen et al., (2011) included two investigations in their reports. Shen et al., (2002) included three investigations.

3. Excluded studies

Duplicate

[1] Kang MY, Gao YM, Huo HQ *et al.* Epidemiological features of chronic and Alzheimer's diseases in the community-based elderly living in cities and counties in Hebei province. *Zhonghua liu xing bing xue za zhi.* 2011;**32**:672-5. (Duplicate with **[57]**)

[2] Li F, Ma Y, Chen R *et al.* Self Assessment Condition, Social Support and the Prevalence of Dementia among the Elderly in a Rural Community of Anhui Province. *Chinese Journal of Disease Control & Prevention* 2011;**1**:19-21. (Duplicate with **[2]**)

[3] Ge X, Wang M, Tang J *et al.* Prevalence of dementia and risk factors in Changsha. *Chin J Nerv Ment Dis* 2014;**8**:493-6. (Duplicate with **[81]**)

[4] Ding D, Zhao Q, Guo Q *et al.* Prevalence survey of dementia among elderly in a urban community in Shanghai. *Chin J Clin Neurosci* 2013;**21**:19-25. (Duplicate with **[75]**)

[5] Ma Y, Jiang Z, Wang J *et al.* Prevalence of dementia and subtypes in Xujiahui, Shanghai. *Chinese Journal of Gerontology*. 2013;**33**:1365-6. (Duplicate with **[76]**)

[6] Jia W, Ma Y, Chen R *et al.* Prevalence and influence factors of dementia: a study on the elderly in some urban and rural communities in China. *Acta Universitatis Medicinalis Anhui* 2012;**8**:944-7. (Duplicate with **[55]**)

[7] Shi Z, Zhang Y, Yue W *et al.* Prevalence and clinical predictors of cognitive impairment in individuals aged 80 years and older in rural China. *Dement Geriatr Cogn Disord* 2013;**36**:171-8. (A sub-sample of **[84]**)

[8] Wang G, Pei G, Xie R *et al.* Epidemiological status investigation of senile dementia in the people aged 65 and older of Tianshui city. *Medical Journal of Chinese People's Health* 2016;**28**:52-55. (A sub-sample of **[87]**)

Non population-based studies

[1] Liu C, Ye D. Prevalence of dementia and risk factors in urban and rural areas. *Healthy People* 2014;**12**:34.

[2] Yu Y, Zhang N, Dong J *et al.* Prevalence of dementia among the elderly and its influential factors. *Chinese General Practice* 2012;**23**:2685-7.

[3] Yu R, Chau PH, McGhee SM *et al.* Trends in prevalence and mortality of dementia in elderly Hong Kong population: projections, disease burden, and implications for long-term care. *Int J Alzheimers Dis* 2012;**2012**:406852.

[4] Pei J-J, Giron MST, Jia J, Wang H-X. Dementia studies in Chinese populations. *Neuroscience bulletin* 2014;**30**:207-16.

[5] Chuang CS, Lin CL, Lin MC *et al.* Decreased prevalence of dementia associated with statins: a national population-based study. *Eur J Neurol* 2015;**22**:912-8.

Unclear diagnostic criteria or methods

[1] Chan C, Xu N, Ma I *et al.* Epidemiological investigation of Alzheimer's disease and cognitive impairment and risk factors. *Journal of Ningxia Medical University* 2014;**3**:320-3.

[2] Chung W. Risk factors for dementia and prevention in rural areas. *Seek Medical And Ask The Medicine* 2012;**8**:468-470.

[3] Zhu R. Situation of elderly patients with Alzhemer's disease and its impact factor in Ninghai region. *China*

Morden Doctor 2012;**8**:16-7.
[4] Chen I, Li J, Wang Z *et al*. Epidemiological investigation of senile dementia in Nanchang. *Jiangxi Medical Journal* 2013;**48**:837-8.
[5] Sun H. Prevalence of senile dementia and risk factors. *China Health Care & Nutrition* 2013;**23**:1777
[6] Wang Y, Zhai J, Zhu F *et al*. Prevalence of Alzheimer's disease and its influential factors among elderly people in communities. *Chin J Public Health* 2011;**7**:827-8
[7] Zhang W. Investigation of dementia prevalence and risk factors in Dongyang city. *Chin J of PHM* 2013;**29**: 237-238
[8] Chu A, Liang X, Chen Y *et al*. Prevalence and risk factors of mild cognitive impairment and dementia in community elderly. *Chinese Journal of Clinical Neurosciences* 2015;**23**:673-677.
[9] Liu Z, Huang Y, Chen H. Descriptive epidemiological study of mental disability attributed to dementia in the people aged 55 years and over in China. *Chinese Mental Health Journal* 2016;**30**:683-688

Focused on Alzheimer's disease or specific subtypes
[1] Huang C, Chen S, Zhao Y *et al*. Prevalence of Alzheimer's disease and risk factors in Shenzhen community-based older people. *Chinese Journal of Practical Nervous Diseases* 2014;**13**:50-2.
[2] Chen S, Li X, Chen J *et al*. Epidemiology of Alzheimer's disease and risk factors. *China Health Industry* 2014;**28**:72-3.
[3] Sun Z, Cui C, Feng H *et al*. Research on Prevalence of Alzheimer's Disease of Fulaerji District in Hei Long Jiang Province. *China Journal of Health Psychology* 2011;**3**:278-80.
[4] Huriletemuer H, Wen S, Zhang C *et al*. An epidemiological study of Alzheimer's disease in elderly Mongolian and Han populations living in rural areas of Inner Mongolia. *Aging Clinical and Experimental Research* 2011;**23**:470-5.
[5] Zou Z, Chen L, Zho L *et al*. Survey on prevalence of Alzheimer's disease in Haining city. *Chinese Journal of General Practice* 2014;**10**:1639-41.
[6] Huriletemuer H, Zhang C, Zhao S *et al*. Study of Alzheimer disease prevalence of Mongolian and Han populations in Inner Mongolia. *J Clin Neurol* 2013;**26**:1-4.
[7] Liao J, Huang HL, Yan J *et al*. The prevalence of Alzheimer's disease in Nanchang community and its influencing factors. *Chinese Journal of Gerontology* 2015;**24**:7176-7177.
[8] Huang F, Shang Y, Lou Y *et al*. Lower Prevalence of Alzheimer's Disease among Tibetans: Association with Religious and Genetic Factors. *J Alzheimers Dis* 2016;**50**:659–667.
[9] Xiao S, Lewis M, Mellor D. The China longitudinal ageing study: overview of the demographic, psychosocial and cognitive data of the Shanghai sample. *J Ment Health* 2016;**25**:131–136

4. Included in the earlier review (Wu et al., 2013)

[1] Chen R, Ma Y, Wilson K *et al*. A multicentre community-based study of dementia cases and subcases in older people in China--the GMS-AGECAT prevalence and socio-economic correlates. International journal of geriatric psychiatry. 2012;**27**:692-702.
[2] Kang M, Gao Y, Huo H *et al*. Prevalence of Alzheimer's disease and its related factors among elderly people

in Hebei province. *Chin J Public Health* 2011;**9**:1123-5.
[3] Fan Q, Zheng J. Investigation of prevalence of Alzheimer and its risk factors. *Modern Preventive Medicine* 2011;**38**:3029-30
[4] Ye GC, Chen FR, Shen KZ. Dementia in people over 60 years old and related risk factors. *Chinese Rural Health Service Administration* 2011;**31**:152-154.
[5] Li L, Li F, Ma Y *et al*. Prevalence of dementia among the elderly in rural community of Anhui. *Chin J Dis Control Prev* 2011;**15**:292-294.
[6] Sun Z, Cui GC, Han YF, Wei HL. Prevalence of Alzheimer's disease among residents of Fulaerji district of Qigihar city, Heilongjiang province. *Chin J Public Health* 2012;**28**:151-153.
[7] Lai M, Zhang H, Yi X *et al*. Epidemiological investigation of Alzheimer's disease in Hainan. *Chin J Geriatr* 2011;**31**:4016-4018.

Appendix S2. Characteristics and quality of included studies (N=96)

Study	Study area	Year of investigation	Age range	Study design	Sampling methods	Response rate	Sample size	Screening tool	Diagnostic criteria	Assessment tool for diagnosis
Ye, 2011 **[1]**	Zhejiang		60+	Two-stage	Cluster-based	95.1%	2445	BSSD, CSI-D	DSM-IV-R	Neuropsychological tests
Li, 2011 **[2]**	Anhui	2008	65+	Two-stage	Unknown	88.3%	1075	CSI-D, 10/66	CCMD-3	
Zhou, 2010 **[3]**	Shanghai	1997-1998	55+	Two-stage	Cluster-based	93.5%	15910	CMMSE	DSM-IV	ADL, POD, FOM, VFT, BD, DS, HAMD, HIS
Ji, 2010 **[4]**	Shanghai	2008	65+	Two-stage	Cluster-based		15158	MMSE, ADL	DSM-IV	
Wang, 2010 **[5]**	Shandong		60+	Two-stage	Individual-based		1500	MMSE	CCMD-3R, DSM-IV, ICD-10	ADL
Chen, 2009 **[6]**	Fujian	2006-2007	60+	Two-stage	Cluster-based	87.2%	2373	MMSE, CES-D	CCMD-3, DSM-IV	FOM, VFT, POD, WAIS, DS, CDT, medical history
Li, 2009 **[7]**	Fujian	2007	65+	Two-stage	Cluster-based	91.9%	2696	MMSE, ADL, CES-D	DSM-IV	HIS, FOM, POD, DS, RVR, WISC
Wang, 2009 **[8]**	Shandong	2008	60+	Two-stage	Cluster-based	96.7%	618	MMSE	DSM-IV	CT/MRI
Rodriguez, 2008 **[9]**	Beijing	1999	65+	One-stage	Cluster-based		2162		10/66 criteria	
Wei, 2008 **[10]**	Hebei	2006	60+	Two-stage	Individual-based		2308	MMSE, ADL	CCMD-2-R	
Yan, 2008 **[11]**	Beijing	2004	65+	Two-stage	Cluster-based	74.2%	1160	CSI-D, 10/66	ICD-10	
Huang, 2007 **[12]**	Guizhou	2005	60+	Two-stage	Cluster-based	81.5%	3229	MMSE, ADL	DSM-IV	HIS, FOM, POD, DS, RVR, CDT, medical history
Tan, 2007 **[13]**	Hubei	2007	55+	Two-stage	Cluster-based		3908	MMSE, ADL	DSM-III-R	HIS, FOM, POD, DS, RVR, BD, HAMD
Tang, 2007	Guangdong	2001-2002	55+	Two-stage	Individual-based	93.2%	5276	MMSE, ADL, CES-D	DSM-IV	HIS, FOM, POD, DS, RVR,

[14]										WISC, HAMD
Zhang, 2006 [15]	4 cities	1997-1998	55+	Two-stage	Cluster-based	94.4%	34807	CMMSE, ADL, medical history	DSM-IV, ICD-10	HIS, FOM, POD, DS, BD, HAMD, VFT, CDT
Zhou, 2006 [16]	Henan		50+	Two-stage	Unknown	74.8%	16095	CMMSE, ADL, medical history	DSM-IV	FOM, POD, DS, BD, CES-D, Animal naming test, HIS
Tang, 2005 [17]	Sichuan	1997-1998	55+	Two-stage	Cluster-based	96.3%	5353	MMSE, ADL	DSM-III-R	HIS, FOM, POD, DS, RVR, BD, HAMD
Tang, 2005 [17]	Sichuan	2000-2001	55+	Two-stage	Cluster-based	90.6%	3908	MMSE, ADL	DSM-III-R	HIS, FOM, POD, DS, RVR, BD, HAMD
Wang, 2002 [18]	Guangdong		60+	Two-stage	Individual-based		1524	MMSE	DSM-IV	HIS, ADL, CT/MRI, medical history
Tang, 2002 [19]	Beijing	1997	60+	Two-stage	Cluster-based	81.6%	2788	MMSE	DSM-IV	FOM, POD, DS, RVR, CDT, VF, medical history
Shen, 2002 [20]	Shanghai	1998	60+	Two-stage	Cluster-based	89.7%	2038	MMSE	DSM-III-R	HIS, medical history
Shen, 2002 [20]	Shanghai	1999	60+	Two-stage	Cluster-based	93.1%	2184	MMSE	DSM-III-R	HIS, medical history
Shen, 2002 [20]	Shanghai	2000	60+	Two-stage	Cluster-based	95.1%	2237	MMSE	DSM-III-R	HIS, medical history
Zhou, 2002 [21]	Chongqing	2001	65+	Two-stage	Cluster-based	87.4%	1519	MMSE, medical history	DSM-IV	HIS, neuropsychological test, medical history
Zhang, 2001 [22]	Beijing	1997-1998	55+	Two-stage	Cluster-based	96.1%	5743	MMSE, ADL	DSM-IV	HIS, FOM, POD, DS, RVR, BD, HAMD, CDR, CDT
Qu, 2001 [23]	Shaanxi	1997-1998	55+	Two-stage	Cluster-based	93.9%	4850	MMSE, ADL	DSM-IV	HIS, FOM, POD, DS, RVR, BD, HAMD,CT/MRI
Sun, 2001 [24]	Liaoning	1999	60+	Two-stage	Individual-based	100%	2000	MMSE, HDS	DSM-IV	HIS, CT/MRI
Fei, 2001 [25]	Shanghai	1999	55+	Two-stage	Unknown	91.3%	462	MMSE	DSM-III-R	HIS, medical history
Lai, 2000	Guangdong		70+	Two-stage	Individual-based	80.5%	3825	CMMSE, ADL, POD,	DSM-III-R	FOM, WAIS, DS, BD, Boston

**[26]**								BDS, VFT		name test, VFT
Wang, 2000 **[27]**	Beijing	1995	60+	Two-stage	Cluster-based	84.1%	5003	MMSE	DSM-III-R, ICD-10	HIS, Neurological examination
Zhang, 2000 **[28]**	Shanghai	1999	55+	Two-stage	Cluster-based	87.7%	1186	MMSE, ADL	DSM-III-R	FOM, POD, DS, RVR, BD
Fan, 2000 **[29]**	Jiangsu	1999	60+	Two-stage	Cluster-based		3268	MMSE, HDS	DSM-III-R	HIS, ADL, ROSEN
Li, 1999 **[30]**	Beijing	1997	60+	Two-stage	Cluster-based	93.5%	1593	MMSE, CRBRS, GDS	ICD-10	HIS, ADL, HAMD, DDDS
Li, 1999 **[31]**	Beijing	1994-1995	60+	Two-stage	Cluster-based	90.3%	1027	MMSE	DSM-III-R	HIS
Xiao, 1999 **[32]**	Hunan	1997-1998	55+	Two-stage	Cluster-based	87.8%	3287	MMSE	DSM-IV	HIS, ADL, FOM, POD, WAIS, RVR, medical history
Wang, 1999 **[33]**	Anhui	1998	65+	Two-stage	Cluster-based	92.0%	2749	HDS	CCMD-2-R, ICD-10	HIS
Tang, 1999 **[34]**	Sichuan	1994	65+	Two-stage	Cluster-based		5987	MMSE, HDS	DSM-III-R	HIS, SDSS
Chiu, 1998 **[35]**	Hong Kong	1995	70+	Two-stage	Individual-based	68.8%	1034	MMSE, GDS	DSM-IV	CT, physical examination, CAMDEX
Lou, 1998 **[36]**	Shanghai	1996-1997	50+	Two-stage	Cluster-based	88.2%	2316	MMSE	DSM-III-R	HIS
Lin, 1998 **[37]**	KaoKaoPing	1993	65+	Two-stage	Individual-based	72.6%	2915	MMSE, BDRS, CES-D, medical history	ICD-10	HIS, ADL, HAMD, CDR, CERAD, BDRS, mental activity
Yu, 1998 **[38]**	Guangdong	1995	65+	Two-stage	Individual-based	98.7%	1018	HDS	CCMD-2-R, ICD-10	HIS, GBS
Wang, 1998 **[39]**	Shandong	1998	60+	Two-stage	Unknown	89.8%	1448	MMSE	CCMD-2, DSM-III-R	HDS, ADL, medical history
Lv, 1998 **[40]**	Zhejiang	1995-1996	60+	Two-stage	Cluster-based	86.1%	1689	BSSD	RDCD	HIS, ADL, FOM, POD, DS, RVR, HAMD, medical history

Chen, 1998 **[41]**	Xinjiang	1995	50+	Two-stage	Cluster-based		2687	HDS	DSM-III	WAIS, CT
Liu, 1998 **[42]**	Kinmen	1993-1994	65+	One-stage	Cluster-based	84.5%	1736	Self-designed questionnaire, CDR, neuropsychological tests	DSM-III-R	HIS, CASI
Xue, 1997 **[43]**	Guangdong	1991-1993	60+	Two-stage	Individual-based	77.1%	3285	MMSE	DSM-III-R	HIS
Wu, 1996 **[44]**	Hainan		60+	Two-stage	Unknown		2528	MMSE	CCMD-2	
Zhang, 1996 **[45]**	Hainan	1992	55+	Two-stage	Cluster-based	95.0%	15164	CMMSE, Functional activity	DSM-III-R	HIS
Liu, 1996 **[46]**	Kaohsiung	1992	65+	Two-stage	Individual-based	84.7%	1016	CMMSE, BDS	DSM-III	HIS, HAMD, CDR, BDS, CERAD, neuropsychological test
Wang, 1995 **[47]**	Shanghai	1992	55+	Two-stage	Individual-based	83.4%	1515	CMMSE, BSSD	DSM-III	ADL, FOM, POD, DS, RVR, Medical history
Wen, 1995 **[48]**	Hainan	1995	55+	Two-stage	Unknown		5465	CMMSE	DSM-III-R	HIS
Gao, 1994 **[49]**	Hunan	1991	60+	Two-stage	Cluster-based	85.4%	5125	MMSE, CRBRS	DSM-III	HIS, HDS, DDDS
Mao, 1993 **[50]**	Fujian	1992	60+	Two-stage	Cluster-based	92.6%	1982	MMSE	DSM-III-R	
Gao, 1993 **[51]**	Shanghai, Jiangsu	1990	60+	Two-stage	Cluster-based	82.2%	3779	MMSE	DSM-III-R	HIS, medical history
Li, 1989 **[52]**	Beijing	1986	60+	Two-stage	Cluster-based	81.9%	1090	MMSE, CRBRS	DSM-III	GMS, DDDS
Zhang, 1989 **[53]**	Shanghai	1987	55+	Two-stage	Cluster-based	82.9%	5055	MMSE	DSM-III	ADL, FOM, POD, DS, RVR, BD, HDS, BDS
Gao, 1989	Beijing	1986	60+	Two-stage	Cluster-based	90.4%	906	CRBRS, neuro-system	DSM-III,	CT

**[54]**								test, self-designed questionnaire	ICD-9	
Chen, 2012 **[55]**	Anhui	2001-2003	65+	One-stage	Cluster-based	94.8%	2917		GMS-AGECAT	
Fan, 2011 **[56]**	Shanxi	2008	60+	Two-stage	Cluster-based		1826	MMSE, ADL	DSM-IV	RVR, CDT, neuropsychological tests
Kang, 2011 **[57]**	Hebei	2010	60+	Two-stage	Cluster-based	88.5%	3632	MMSE	DSM-IV	HIS, ADL
Chen, 2012 **[55]**	4 province	2008-2009	65+	One-stage	Cluster-based	93.8%	3227		GMS-AGECAT	
Chen, 1992 **[58]**	Beijing	1986	60+	Two-stage	Cluster-based	85.0%	5172	MMSE, CRBRS	DSM-III	GMS, DDDS, medical history
Yip, 1992 **[59]**	Taipei	1990	65+	Two-stage	Individual-based	89.4%	1038	CMMSE, ADL	DSM-III-R	HIS, IADL, Memory and Behavior Problem Checklist, CMMSE, simplified high cortical function examination
Lee, 1997 **[60]**	Ilan		65+	Two-stage	Cluster-based	92.2%	2717	CMMSE	DSM-III-R	Neuropsychological tests
Yu, 1994 **[61]**	Shaanxi	1994	65+	Two-stage	Cluster-based	82.2%	2422	GHQ	DSM-III-R	HIS, MMSE, HDS-R, CT, Medical history
Sun, 2012 **[62]**	Heilongjiang	2008-2009	60+	Two-stage	Cluster-based	92.8%	3698	CMMSE	DSM-IV	HIS, ADL, HAMD
Pong, 2010 **[63]**	Gansu	2007-2008	65+	Two-stage	Individual-based	77.1%	569	MMSE, HDS	DSM-IV	
Li, 2008 **[64]**	Hebei	2003	65+	Two-stage	Individual-based	98.5%	2126	MMSE, BSSD	DSM-IV	
Yuan, 2005 **[65]**	Jiangxi	2002	60+	Two-stage	Cluster-based		2126	CRBRS, CIDI	CCMD-3, ICD-10	ADL, medical history, CIDI
Liang, 2003 **[66]**	Guangdong	2002	60+	Two-stage	Cluster-based	89.6%	1418	MMSE	DSM-IV	HIS, ADL, FOM, HDS, DS, RVR, Medical history

Wu, 2003 **[67]**	Hainan	2003	60+	Two-stage	Cluster-based		9770	MMSE	DSM-III-R	HIS
Tang, 1998 **[68]**	Beijing	1996	60+	Two-stage	Cluster-based	97.1%	501	MMSE, BDS, CES-D	DSM-III-R, ICD-10	CRBRS, CT
Chen, 2004 **[69]**	Hainan	2002	60+	Two-stage	Individual-based		12628	MMSE	DSM-III-R	HIS
Cheng, 1989 **[70]**	Shanghai		60+	Two-stage	Cluster-based	96.8%	1953	MMSE, HDS, SPMSQ	DSM-III	
Lai, 2011 **[71]**	Hainan	2010	55+	Two-stage	Cluster-based		7665	HDS	CDR	HIS
Liu, 1995 **[72]**	Taiwan	1994	60+	Two-stage	Cluster-based	82.6%	2288	MMSE	DSM-III-R	HIS, BDS
Ho, 2012 **[73]**	Jiangxi	2011	65+	Two-stage	Cluster-based	96.0%	1029	10/66 assessment tool	CCMD-3	
Sun, 2012 **[74]**	Shanghai	2010-2011	60+	Two-stage	Cluster-based	71.7%	1472	CMMSE	DSM-IV-TR	ADL, CSDD, CT/MRI
Ding, 2013 **[75]**	Shanghai	2010	60+	One-stage	Individual-based	69.5%	3141		DSM-IV	MMSE, CDR, ADL, CES-D, neuropsychological battery
Ma, 2013 **[76]**	Shanghai		65+	Two-stage	Individual-based	95.8%	2442	CMMSE, ADL	DSM-IV, ICD-10	FOM, POD, HDS, WAIS, HAMD
Wang, 2014 **[77]**	Guangdong	2011	60+	Two-stage	Cluster-based		2338	CMMSE, ADL	DSM-IV	
Wang, 2014 **[78]**	Zhejiang	2010	60+	Two-stage	Cluster-based	90.8%	1906	CMMSE	ICD-10	WAIS, medical history
Li, 2014 **[79]**	Zhejiang	2013	60+	Two-stage	Individual-based		2451	CMMSE, ADL	CCMD-2-R	
Mong, 2014 **[80]**	Xinjiang	2010-2012	55+	Two-stage	Individual-based	98.6%	3610	CMMSE, self-designed questionnaire	DSM	ADL, FOM, DS, RVR, CDR, CDT, CT/MRI
Tang, 2014	Hunan	2011-2012	55+	Two-stage	Cluster-based	99.4%	10026	MMSE, ADL	DSM-IV-TR	

**[81]**										
Li, 2015 **[82]**	Guangxi	2012-2014	60+	Two-stage	Unknown	79.4%	889	CMMSE	DSM-IV-TR	ADL, CDT, FOM, RVR, CT
Jia, 2014 **[85]**	Five areas	2008-2009	65+	One-stage	Cluster-based	74.4%	10276		DSM-IV	HIS, FAQ, MoCA, MMSE, CDR, medial history, CT/MRI
Sun, 2014 **[83]**	Taiwan	2008-2011	65+	One-stage	Individual-based	36.5%	10432		NIA-AA	CDR, TMSE
Lam, 2006 **[86]**	Hong Kong	2005-2006	60+	Two-stage	Individual-based	88.5%	6100	CMMSE, AMIC	DSM-IV	
Ji, 2015 **[84]**	Tianjin	2011-2012	60+	Two-stage	Cluster-based	97.1%	5578	CMMSE	DSM-IV	
Yang, 2016 **[90]**	Zhejiang	2014	65+	One-stage	Cluster-based	97.7%	2015		NIA-AA	CDR, MMSE
Li, 2015 **[91]**	Tianjin		60+	Two-stage	Cluster-based		2532	MMSE, ADL	DSM-IV	HIS, HAMD
Lou, 2016 **[88]**	Guangdong		60+	One-stage	Cluster-based		3224		ICD-10	CMMSE, ADL
Zhang, 2015 **[89]**	Heilongjiang		60+	Two-stage	Cluster-based		2761	MMSE	DSM-IV	ADL
Wang, 2016 **[87]**	Gansu	2014-2015	60+	Two-stage	Individual-based	99.4%	2416	HDS, GHQ	DSM-IV	CDR, HIS
Gong, 2002 **[92]**	Hainan	2001	60+	Two-stage	Cluster-based	92.2%	2961	MMSE, CRBRS	CCMD-3-R	DDDS, HIS

ADL: Activities of Daily Living; AMIC: Abbreviated Memory Inventory for Chinese; BD: Block design of Wechsler Intelligence Scale for Children; BDS: Blessed Dementia Scale; BSSD: Behavioral Syndromes Scale for Dementia; CASI: Cognitive Abilities Screening Instrument; CAMDEX: Cambridge Mental Disorders of the Elderly Examination; CCMD: Chinese classification of mental disorders; CDR: Clinical Dementia Rating; CDT: Clock Drawing Test; CERAD: Consortium to Establish a Registry for Alzheimer's Disease; CES-D: Center for Epidemiologic Studies Depression Scale; CIDI: Composite International Diagnostic Interview; CMMSE: Chinese Mini-Mental State Examination; CRBRS: Crichton Royal Behaviour Rating Scale; CSDD: Cornell scale for depression in dementia; CSI-D: Community Screening Instrument for Dementia; CT/MRI: Computed tomography or magnetic resonance imaging; DDDS: Dementia differential diagnostic schedule; DS: Digit Span test; DSM: Diagnostic and Statistical Manual of Mental Disorders; FAQ: Functional Activities Questionnaire; FOM: Fuld object-memory Evaluation; GBS: Gottfries ,Brane and Steen Scale; GDS:

Geriatric Depression Scale; GHQ: General Health Questionnaire; GMS: Geriatric Mental Status; HAMD: Hamilton Rating Scale for Depression; HDS: Hasegawa's Dementia Scale; HIS: Hachinski Ischemic Scale; ICD: The International Statistical Classification of Diseases and Related Health Problems; MoCA: Montreal Cognitive Assessment; NIA-AA: criteria of the National Institute on Aging-Alzheimer's Association; POD: Pfeiffer Outpatient Disability Questionnaire; RVR: Rapid Verbal Retrieve; SPMSQ: Pfeiffer's short portable mental status questionnaire; TMMSE: Taiwanese Mini-Mental State Examination; WAIS: Wechsler Adult Intelligence Scale

Study quality assessment

The quality of all included studies was assessed using the methods reported in the World Alzheimer Report.[1,2] A summarised score for study quality was calculated based on: (1) sample size: 0 point for 499 or below, 0.5 point for 500–1499, 1 point for 1500–2999 and 2 points for 3000 or above; (2) study design: 0 point for two-stage study without sampling of screen negatives, 1 point for two-stage study with sampling of screen negatives but no weighting back, 2 points for one-stage or two-stage study with appropriate sampling and weighting; (3) response rate: 0 point for unknown, 1 point for <60%, 2 points for 60–79%, 3 points for >80%; and (4) diagnostic assessment: 1 point each for multi-domain cognitive test battery, formal disability assessment, informant interview and clinical interview.

Results of study quality assessment are reported in Table S2-1. The mean score for study quality was 6.5 (Std.: 1.9) with a range between 2.5 and 10.0. Variation in prevalence estimates was not associated with study quality (meta-regression coefficient 0.00; 95% CI: -0.34, 0.33) and I-square remained to be high (98.3%) when adjusting for study quality. Scores for study quality did not change considerably over time. This indicates more recent studies did not show better quality of study methods compared to earlier studies.

Table S2-1: Results of study quality assessment

Item	N (%)
Sample size	
<500	1 (1.0)
500-1499	17 (17.7)
1500-2999	41 (42.7)
>3000	37 (38.5)
Study design	
One-stage	9 (9.4)
Two-stage	82 (85.4)
Multistage with false negative correction and weights	5 (5.2)
Response rate	
Unknown	21 (21.9)
<60%	1 (1.0)
60-79%	10 (10.4)
≥80%	64 (66.7)
Diagnostic assessment	
Multi-domain cognitive battery, informant interview, a formal assessment of disability and a clinical interview	9 (9.4)
Overall score	
Mean (std.)	6.5 (1.9)
Time periods	
Before 1990	7.2 (1.8)
1990-1994	6.6 (1.7)
1995-1999	6.6 (1.9)
2000-2004	6.2 (2.2)
2005-2009	6.4 (1.9)
2010-2015	6.4 (2.2)

References

1. Alzheimer's Disease International. *World Alzheimer Report 2015: the global impact of dementia: an analysis of prevalence, incidence, cost and trends*. London: Alzheimer's Disease International, 2015.

2. Prince M, Bryce R, Albanese E, Wimo A, Ribeiro W, Ferri CP. The global prevalence of dementia: a systematic review and metaanalysis. *Alzheimers Dement* 2013;**9**:63-75.

Appendix S3. Estimation on number of people with dementia

To estimate the number of population with dementia and take into account methodological factors and geographical variation, age-stratified prevalence was calculated based on the results of meta-regression modelling. Since the pooled estimates of crude prevalence by five-year age groups approximately followed the pattern of doubling every 5 years, it was assumed that:

(1) P= prevalence of dementia in people aged 60 or over; predicted estimates from meta-regression model adjusting for methodological factors, geographical areas and time periods

(2) p_1= prevalence of age 60-64

p_2= prevalence of age 65-69

p_3= prevalence of age 70-74

p_4= prevalence of age 75-79

p_5= prevalence of age 80-84

p_6= prevalence of age 85-89

p_7= prevalence of age 90 or over

(3) The pooled estimates for prevalence of dementia approximately doubled every 5 years:

$p_2=2p_1$; $p_3=4p_1$; $p_4=8p_1$; $p_5=16p_1$; $p_6=32p_1$; $p_7=64p_1$

Based on (1)-(3), age-stratified prevalence of dementia can be calculated as

$p_1=\frac{P}{\sum_{i=1}^{7} 2^{(i-1)} \times r_i}$ where r_i= the proportion of the age group in the study population

$p_{2\sim7}$ can be calculated using p_1 and (3)

Figure S3-1 shows results of age-stratified prevalence estimates by different diagnostic criteria. Patterns of estimated and crude prevalence were generally similar across age groups.

Figure S3-1: Estimated and crude prevalence by age groups and diagnostic criteria

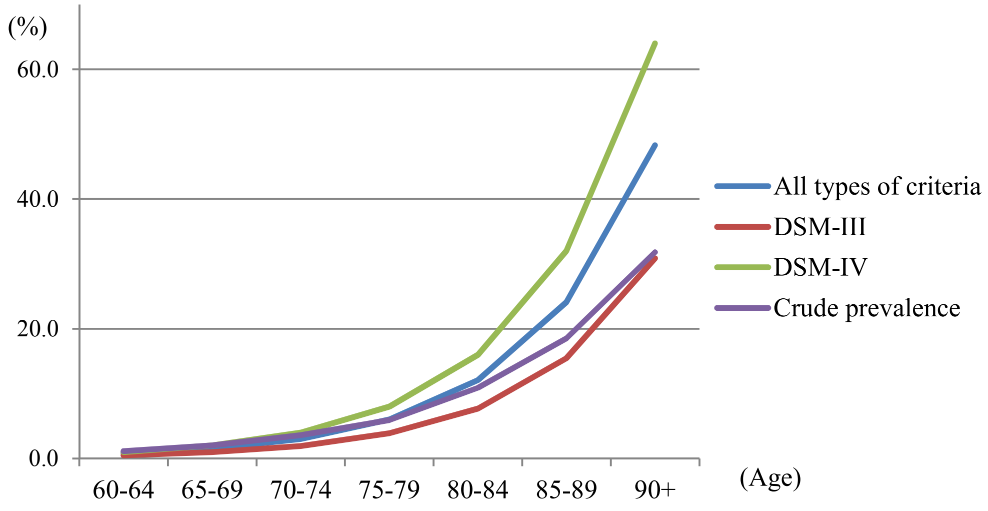

Regional prevalence in people aged 60 or above was estimated based on the results of meta-regression model including methodological factors, geographical areas and time periods. Since most studies in Hong Kong and Taiwan focused on the population aged 65 or above, the estimation was only generated from age 65. Since studies from western areas did not report prevalence estimate in age 90 or above, the estimate was calculated for age 85 or above. Using the same method, Figure S3-2 shows age-stratified prevalence across the five areas.

Figure S3-2: Estimated prevalence by five-year age groups and geographical areas

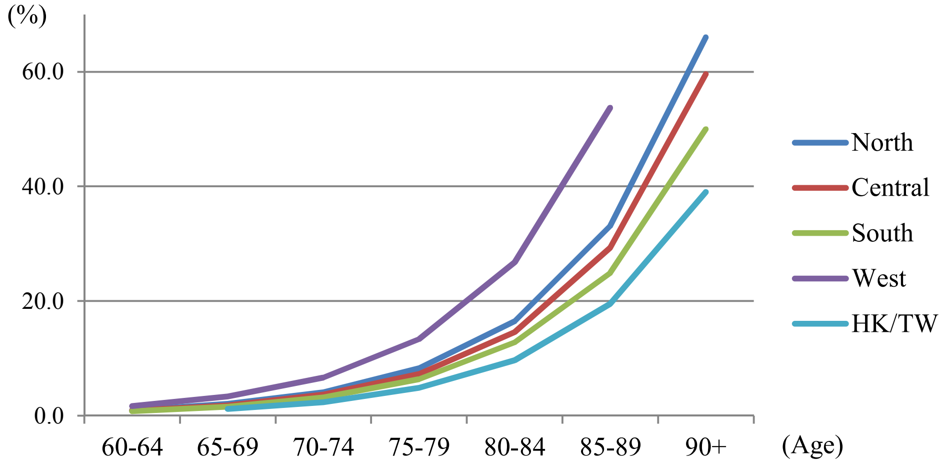

The five-year prevalence estimates were applied to population structures in provinces and cities of China, Hong Kong and Taiwan (Figure S3-3). Information on age structures was obtained from the National Bureau of Statistics for China, Department of Interior for Taiwan and Census and Statistics Department for Hong Kong Special Administrative regions (SAR).

Figure S3-3: Estimated number of people with dementia in the population aged 60 or above by provinces and cities in China and Taiwan

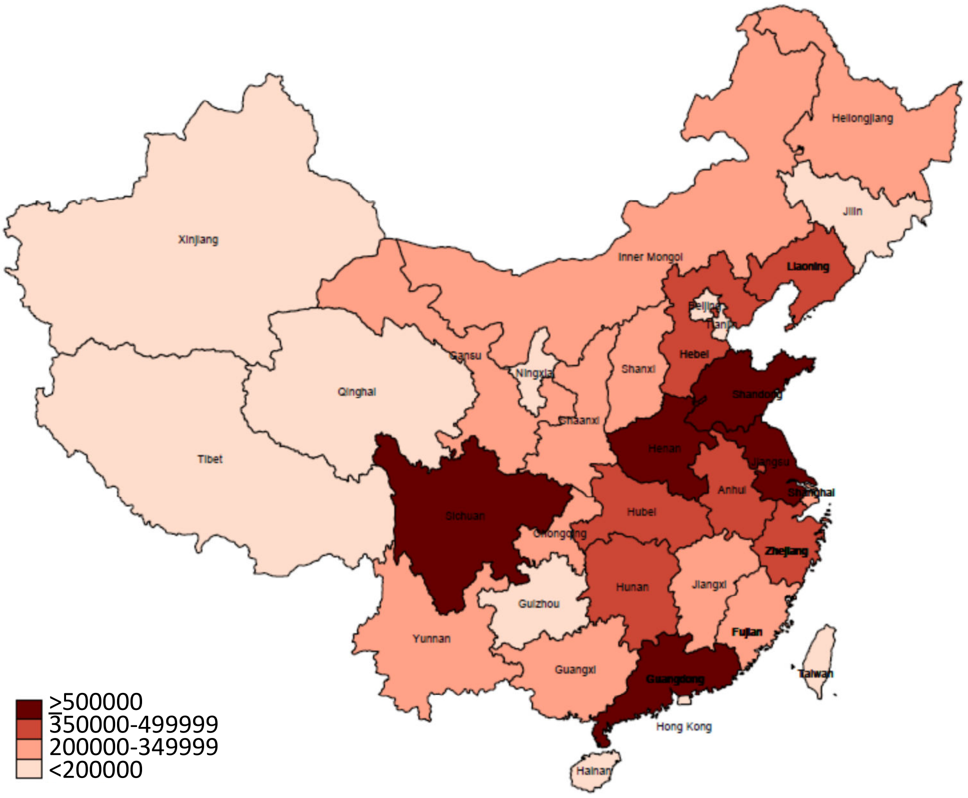

Appendix S4. PRISMA checklist

Section/topic	#	Checklist item	Reported on page #
**TITLE**			
Title	1	Identify the report as a systematic review, meta-analysis, or both.	p.1
**ABSTRACT**			
Structured summary	2	Provide a structured summary including, as applicable: background; objectives; data sources; study eligibility criteria, participants, and interventions; study appraisal and synthesis methods; results; limitations; conclusions and implications of key findings; systematic review registration number.	p.3-4
**INTRODUCTION**			
Rationale	3	Describe the rationale for the review in the context of what is already known.	p.6-7
Objectives	4	Provide an explicit statement of questions being addressed with reference to participants, interventions, comparisons, outcomes, and study design (PICOS).	p.7
**METHODS**			
Protocol and registration	5	Indicate if a review protocol exists, if and where it can be accessed (e.g., Web address), and, if available, provide registration information including registration number.	N/A, systematic review protocol is provided in S1.
Eligibility criteria	6	Specify study characteristics (e.g., PICOS, length of follow-up) and report characteristics (e.g., years considered, language, publication status) used as criteria for eligibility, giving rationale.	p.8, S1

Information sources	7	Describe all information sources (e.g., databases with dates of coverage, contact with study authors to identify additional studies) in the search and date last searched.	p.8-9, Fig 1, S1
Search	8	Present full electronic search strategy for at least one database, including any limits used, such that it could be repeated.	Fig 1, S1
Study selection	9	State the process for selecting studies (i.e., screening, eligibility, included in systematic review, and, if applicable, included in the meta-analysis).	p.8, Fig 1
Data collection process	10	Describe method of data extraction from reports (e.g., piloted forms, independently, in duplicate) and any processes for obtaining and confirming data from investigators.	p.9
Data items	11	List and define all variables for which data were sought (e.g., PICOS, funding sources) and any assumptions and simplifications made.	p.9-10
Risk of bias in individual studies	12	Describe methods used for assessing risk of bias of individual studies (including specification of whether this was done at the study or outcome level), and how this information is to be used in any data synthesis.	p.9, S2
Summary measures	13	State the principal summary measures (e.g., risk ratio, difference in means).	p.9
Synthesis of results	14	Describe the methods of handling data and combining results of studies, if done, including measures of consistency (e.g., I^2) for each meta-analysis.	p.10-11
Risk of bias across studies	15	Specify any assessment of risk of bias that may affect the cumulative evidence (e.g., publication bias, selective reporting within studies).	N/A
Additional analyses	16	Describe methods of additional analyses (e.g., sensitivity or subgroup analyses, meta-regression), if done, indicating which were pre-specified.	p.11-12
**RESULTS**			
Study selection	17	Give numbers of studies screened, assessed for eligibility, and included in the review, with reasons for exclusions at each stage, ideally with a flow diagram.	Fig 1, S1

Study characteristics	18	For each study, present characteristics for which data were extracted (e.g., study size, PICOS, follow-up period) and provide the citations.	p.10-13, S2
Risk of bias within studies	19	Present data on risk of bias of each study and, if available, any outcome level assessment (see item 12).	S2
Results of individual studies	20	For all outcomes considered (benefits or harms), present, for each study: (a) simple summary data for each intervention group (b) effect estimates and confidence intervals, ideally with a forest plot.	Fig 2
Synthesis of results	21	Present results of each meta-analysis done, including confidence intervals and measures of consistency.	p.12-14
Risk of bias across studies	22	Present results of any assessment of risk of bias across studies (see Item 15).	N/A
Additional analysis	23	Give results of additional analyses, if done (e.g., sensitivity or subgroup analyses, meta-regression [see Item 16]).	p.13-15
**DISCUSSION**			
Summary of evidence	24	Summarize the main findings including the strength of evidence for each main outcome; consider their relevance to key groups (e.g., healthcare providers, users, and policy makers).	p.15
Limitations	25	Discuss limitations at study and outcome level (e.g., risk of bias), and at review-level (e.g., incomplete retrieval of identified research, reporting bias).	p.16-18
Conclusions	26	Provide a general interpretation of the results in the context of other evidence, and implications for future research.	p.18-21
**FUNDING**			
Funding	27	Describe sources of funding for the systematic review and other support (e.g., supply of data); role of funders for the systematic review.	p.22

From: Moher D, Liberati A, Tetzlaff J, Altman DG, The PRISMA Group (2009). Preferred Reporting Items for Systematic Reviews and Meta-Analyses: The PRISMA Statement. PLoS Med 6(7): e1000097. doi:10.1371/journal.pmed1000097
